# Effects of cognitive load and different exercise intensities on perceived effort in sedentary university students: a follow up of the Cubo Fitness Test validation

**DOI:** 10.3389/fpsyg.2023.1254767

**Published:** 2023-12-08

**Authors:** Gabriele Signorini, Raffaele Scurati, Andrea Bosio, Gloria Maestri, Marta Rigon, Athos Trecroci, Pietro Luigi Invernizzi

**Affiliations:** ^1^Department of Biomedical Sciences for Health, Università degli Studi di Milano, Milan, Italy; ^2^Human Performance Laboratory, Mapei Sport, Olgiate Olona, Italy

**Keywords:** mental fatigue, workload, sedentary lifestyle, CFT, NASA-TLX, cardio-respiratory and muscular endurance, flexibility, core muscular efficiency

## Abstract

Work and intellectually fatiguing environments can significantly influence the health of individuals, which is strictly bound to motor efficiency. In particular, desk workers and university students may have a sedentary lifestyle and a condition of mental fatigue caused by daily routine, which could impair motor efficiency. The assessment is a starting point for enhancing awareness of the individual’s psychophysical condition through the perception of one’s body motor efficiency, motivating to move towards improvement. This way, a submaximal test based on perceived exertion was developed (Cubo Fitness Test, CFT) and validated in previous studies. Hence, two further studies were employed to enhance the consistency and accuracy of this instrument in different conditions. The first study investigated the internal responsiveness of CFT, evaluating if mental fatigue could affect motor efficiency. The second study investigated which perceived intensity (weak, moderate, strong, or absolute maximum) could be more reliable for applying the CFT (as previous research focused the investigation only on moderate intensity). In the first investigation, participants assessed two stimuli (mental fatigue induced with a Stroop color-word task and a neutral condition based on the vision of a documentary) lasting 60 min each. The quality of psychophysical recovery (total quality recovery) and the mood state (Italian Mood State questionnaire) were evaluated before the stimuli. After the fatiguing or the neutral task, the mood state was newly assessed, together with the evaluation of the workload’s characteristics (Nasa TLX) and the CFT motor efficiency. In the second investigation, participants had to perform CFT twice for each at different intensities of Borg’s Scale of perceived exertion. Researchers successfully requested to fill out the NASA TLX questionnaire regarding the perceived workload characteristics of CFT, and the reliability of each intensity was assessed. Results seem to enhance the consistency and the accuracy of the instrument. Indeed, findings evidenced that CFT is not influenced by mental fatigue conditions typical of the intellectual work of desk workers and university students for which this test was specifically conceived. Moreover, moderate and strong perceived intensity are the most adequate conditions to assess motor efficiency in these populations.

## 1 Introduction

Desk workers and university students typically live in physical inactivity and sedentary conditions. These populations are estimated to sit around 10 h per day during working and class activities, respectively (McCrady and Levine, 2009; Clemes et al., 2016; Castro et al., 2020). These sedentary behaviors have been further exacerbated by the COVID-19 pandemic in both populations (Ráthonyi et al., 2021; Dziewior et al., 2022). Sedentary and intellectual working environments have a significant influence on individuals’ health. It suffices to think that sedentary occupations are associated with increased cardiovascular risk and a higher likelihood of developing musculoskeletal diseases (World Health Organization et al., 2018). These occupations are also associated with worse mental health and with an increased risk of developing depression, anxiety and dementia (Teychenne et al., 2010, 2015; Lee and Kim, 2019; Yan et al., 2020). In particular, in the last years, university students are reported to have doubled symptoms of depression and anxiety due to their workload (Prince, 2015). Indeed, as for desk workers, students’ workload is determined not only by the number of study hours (dedicated explicitly to memorizing information and reasoning) but even by the time pressure, which increases stress and negative feelings (Smith, 2019). Given the beneficial effect that adequate levels of physical activity has on psychophysical health, one of the main aims for the health promotion in students and desk-workers population should be the subsistence of adequate levels of motor efficiency, defined as physical and coordinative abilities which determine less effort in producing performance (Herbert et al., 2020). It represents a crucial element to maintain a lifespan physical autonomy (Lay et al., 2002; Raiola et al., 2022). Indeed, the subsistence of adequate levels of conscious physical fitness and economy in movement energy expenditure constitute key elements to keep body awareness, physical autonomy, and high quality of life (Graves and Larkin, 2006; Duda et al., 2014; Furtado et al., 2015; Sumpter et al., 2015; Tornero-Quiñones et al., 2020).

Given these considerations, workplaces and universities must prioritize health promotion and physical activity, supporting individuals to develop a comprehensive understanding of their bodies adopt competent management practices, and maintain adequate motor efficiency (Plotnikoff et al., 2015; World Health Organization, 2018). The assessment is a starting point for enhancing awareness of the individual’s psychophysical condition through perception of one’s body motor efficiency, motivating to move towards improvement. (Invernizzi et al., 2021). Accordingly, relying on valid and practical tests is imperative to assess motor efficiency in people engaged in predominantly sedentary activities.

The Cubo Fitness Test (CFT) is a diagnostic tool developed to assess motor efficiency. The optimal reliability and good criterion validity of CFT with university student and desk-worker populations was previously confirmed by Invernizzi et al. (2021, 2022). The CFT is performed at a sub-maximal intensity (perceived as *moderate* from rating of perceived exertion scales) requiring a short time to be accomplished (without sophisticated and expensive equipment). The index of motor efficiency resulting from the five physical components tested (Cardio-respiratory fitness: Ruffier test; Muscular fitness: push-up and seated sit-up tests; Flexibility fitness: shoulder mobility and chair sit and reach tests) is the primary outcome of the CFT. It provides valuable parameters for possible physical activity interventions, such as the UP150 (workplace physical activity implementation project: “UP” stands for the Italian acronym that can be translated in “proactive office,” and “150” represents the 150 minimum minutes of moderate physical activity recommended by the World Health Organization for sedentary or less active people) project (World Health Organization, 2020; Invernizzi et al., 2021).

Desk workers and university students spend a significant amount of time on cognitive and mental demanding activities (Frone and Tidwell, 2015). Intense cognitive tasks performed for several minutes can lead to increased subjective feelings of tiredness and decreased cognitive performance, also defined as mental fatigue (Marcora et al., 2009). Further studies reported that acute and chronic mental fatigue could induce negative mood while bringing mental and physical health problems, respectively (Smith et al., 2016). More specifically, university students’ mental workload is even demonstrated to influence their sleep and cognitive functions (Rutala et al., 1990; Mizuno et al., 2011).

In the above contexts, in order to improve working conditions and lifestyles, it would be desirable for individuals to be aware of the disadvantages of a sedentary lifestyle in terms of physical and mental condition. This would lead to a change toward healthy daily habits can take place, achieving and maintaining good levels of physical literacy (Whitehead, 2010). In this perspective, the CFT is advantageous because it allows knowledge and, thus, awareness of the physical fitness status of individuals. In addition, it is based on effort perception scales, which guarantee the tested subjects’ safety and prevent occupational injuries and illness. The body perception is particularly favourable for the promotion of physical literacy and exercise educations (Pastor-Cisneros et al., 2021). Indeed, these educations should always consider the individual psychophysical characteristics and act in agreement with actual body condition (Birnbaumer et al., 2022). Only with this awareness, individuals could independently choose appropriate activities through lifespan (Deci and Ryan, 2000; Finch and Owen, 2001; Invernizzi et al., 2022).

Perceived exertion refers to *the perception of how hard, heavy and tiring a specific physical task is* (Marcora, 2010). It mainly refers to the sensations concerning dyspnea and how hard a person drives his/her active limbs. The perception of effort is beneficial as an indicator to monitor the effect of a predetermined exercise based on an external load and to modulate the exercise. This tool can be utilized whether the subject is an athlete or a patient undergoing a rehabilitation or training program. The perception of effort allows immediate feedback on the internal load of an exercise. To do this, numerical or visual scales are used, often assisted by verbal anchors to which the subject can refer, which are very effective in providing a value that reflects the perceived exercise intensity (Razon et al., 2012).

Given these considerations, when conducting a physical performance test to promote physical activity and educate on good practices for health in these intellectual sedentary contexts, it is necessary to consider whether and how much mental fatigue may affect the test outcome. Some studies (Pageaux et al., 2014; Van Cutsem et al., 2017a; Grgic et al., 2022) highlighted that mental fatigue negatively affects physical performance. Mental fatigue can cause changes in technique, decision-making, skill execution. The negative influence on physical performance has mainly been attributed to an increased perception of effort (Smith et al., 2016). Therefore, from these considerations it emerges that the CFT must be able to give objective evaluations in specific sedentary contexts regardless of the fluctuations in mental effort during the different periods of the year (Signorini et al., 2022). Indeed, the CFT should work in specific environments where mental fatigue represents a typical condition of the individuals’ daily routine (Invernizzi et al., 2021).

The CFT protocol assumes that sub-maximal effort (moderate perception of effect) are administered to participants. There are many advantages to using sub-maximal tests (defined as a test that conduct the participant to a predetermined level of intensity compared with and below the maximum capacity; Shushan et al., 2022), including greater safety and the possibility of testing people who are not accostumed to exercise and who find it challenging to tolerate maximal-intensity exertion, making it possible to extend measurements to sedentary participants. Table 1 shows the current status of the CFT validation process.

**Table 1:** Status of the validation process of the Cubo Fitness Test.

Validation process		Status	Publications
Conceptual and measurement model		Investigated	Invernizzi et al. (2021)
Validity	Logical validity	Investigated	Invernizzi et al. (2021)
	Criterion validity	Investigated	Invernizzi et al. (2021)
	Construct validity	To be investigated	Future investigation
Reliability		Investigated	Invernizzi et al. (2021, 2022)
Responsiveness	External	Investigated	Invernizzi et al. (2022)
	Internal	To be investigated	Present publication (Study 1)
Reliability of alternative forms		To be investigated	Present publication (Study 2)

The validation process follows the procedure by Impellizzeri and Marcora (2009).

The present study will progress the CFT validation process. In the first study (Study 1), we investigated the internal responsiveness (defined as the ability of a measure to change over a particular prespecified time frame or condition) stating that an absence of it determines a highest degree of the test’s consistency and accuracy in measuring populations subjected to acute mental fatigue (as desk-workers and university students; Husted et al., 2000). After that, in a second study (Study 2), we examined at which intensity of perceived exertion (*weak*, *moderate*, *strong*, or *absolute maximum*) the CFT shows the highest relative reliability (defined as “degree to which individuals maintain their position in a sample with repeated measurements”; Impellizzeri and Marcora, 2009). Moreover, in this last study, the intensity-induced changes in the perception of test load’s characteristics were further investigated.

## 2 Study 1

Study 1 aims to examine the internal responsiveness of CFT, ensuring that mental fatigue does not affect the CFT results. Considering research by Marcora et al. (2009), which claims that mental fatigue can affect effort perception and physical performance, we wanted to test whether CFT results would be affected by mental fatigue due to intense cognitive load from work or study. For this purpose, participants, after a familiarization period, were subjected to two experimental sessions, lasting 60 min each, with mental fatigue and neutral stimuli in a randomized manner. Before assessing CFT, we measured the participants’ quality of psychophysical condition, mood and task workload.

### 2.1 Methods of study 1

The sample size required for Study 1 was evaluated using the statistical software G*power. It used the T-test category selecting “MEANS: the difference between two dependent means (Matched pairs).” The Cohen’s dz. effect size was set at 1.08, calculated from the outcomes of a previous study that investigated the changes in Cubo Fitness Test results (the principal variable investigated in the present study). The estimated power, 1-β, was set at 0.95 and the alpha value at 0.05. With these inputs, the software indicated 11 participants as adequate sample size (Actual Power = 0.953). We decided to recruit 18 participants to prevent the reduction of the statistical power caused by possible dropouts. The sample comprised 13 male and five female university students 21.6 ± 2.5 years old. The sample can be considered as normal weight (weight: 72.0 ± 11.8 Kg; Height: 1.75 ± 1.0 m; BMI: 23.5 ± 2.7 kg/m^2^). This study included sedentary university students who spent at least 7 h/day studying or attending university lessons while excluded students who regularly practised sports or physical activity during free time. The analysis of physical activity habits was performed using the International physical activity questionnaire (IPAQ), as will be explained later. The study was conducted in accordance with the declaration of Helsinki and was approved by the ethics committee of the University of Milan (14 September 2020, number 84/20).

### 2.2 Procedure

Participants underwent a three-day familiarization period with all tests and questionnaires. After 48 h, they participated in two experimental sessions, including mental fatigue stimulus (MF) and a neutral stimulus (NS), 1 week apart (Penna et al., 2018; Staiano et al., 2019). To avoid unwanted effects due to the sequence of the proposed stimuli, the order of administration of the two stimuli was randomized (using the site www.randomization.com) for each participant.

Participants were recruited after the administration of the IPAQ questionnaire. Before the beginning of the experimental protocol, participants were required to come to the experimental session with sufficient psychophysical recovery, measured using the Total Quality Recovery Scale (TQR). Immediately after the compilation of the TQR, the participants were asked to answer the Italian version of Brunel’s mood state questionnaire (ITAMS). Afterwards, participants received the expected stimulus (MF or NS) for 60 min. At the end of the stimulus, the participants filled out the Nasa TLX questionnaire to evaluate the typology of load created by the proposed stimulus. Finally, the participants performed the Cubo Fitness Test (CFT).

#### 2.2.1 International physical activity questionnaire

The International physical activity questionnaire (IPAQ) is a validated helpful questionnaire to assess the participants’ weekly physical activity (Craig et al., 2003). The candidate must answer the questionnaire about the activities performed the previous week. The questionnaire returns the weekly amount of activity measured in METs. The cut-off suggested by Craig et al. (2003) to define the profile of the participants is (a) sedentary < 700 METs; (b) 700 METs < minimally active <2,519 METs; (c) Highly engaging in physical activity >2,520 METs.

#### 2.2.2 Total quality recovery scale

The Total quality recovery scale (TQR) is a validated scale used to evaluate the psychophysical recovery state (Kenttä and Hassmén, 1998). The scale has been used in previous studies to assess psychophysical recovery before performing the CFT (Invernizzi et al., 2021, 2022). The TQR scale is composed of a range of values from 6 to 20 with verbal anchors based on recovery status (from “very, very poor recovery” to “very, very good recovery”). The participants are asked to select the verbal anchor that better address the psychophysical recovery at a specific moment (soon previous to the execution of the CFT), then the participants indicate a number from the scale close to the chosen term (i.e., participant feels his recovery as good, so he/she indicates the value “15” which is close to the verbal anchor “good recovery”). To begin the experimental procedure, the participant has to indicate a minimum value of reasonable recovery (number 13).

#### 2.2.3 Mental fatigue

To induce the state of mental fatigue (MF), the participants performed a fatiguing stimulus by the modified Stroop colour word task for 60 min (Al-Shargie et al., 2019). The Stroop colour word task is a cognitive computer-based task during which four colour names (blue, green, yellow, and red), coloured differently (i.e., the word “blue” could be randomly displayed coloured in blue, green, yellow or red), are displayed in random sequence. The participant must correctly indicate the colour of the displayed word as fast as possible by pressing the computer’s keyboard keys B, G, Y, and R (for blue, green, yellow and red, respectively). After each selection, the program gives visual feedback indicating the correctness of the answer (i.e., the word “red” is displayed in green. In this case the keyboard key G has to be pressed). Immediately after the visual feedback, a pause of 1.5 s is expected before the next colour name. During the familiarization, the participants performed a version of the test with a reduced time (10 min). The task was performed in a silent and quiet room.

#### 2.2.4 Neutral stimulus

The neutral stimulus (NS) was administrated in a controlled, quiet, silent room. The participants are required to sit and watch a historical documentary on “History Channel” about the Siberian railways. The documentary lasted 60 min, as in the MF (Staiano et al., 2019).

#### 2.2.5 Italian mood state questionnaire

The Italian mood state questionnaire (ITAMS) is the Italian version of Brunel’s mood state questionnaire and aims to assess the participants’ mood state (Quartiroli et al., 2017). It requires compiling a list of 24 questions with a Likert scale ranging from 0 (“not at all”) to 4 (“extremely”), indicating the mood status of the moment. The total score (the sum of scores of all items) and the Total Mood Disturbance (TMD, calculated as follows: TMD = depression + tension + anger + fatigue + confusion +100 – vigor) are computed to completely evaluate the mood status of the participants (Mashiko et al., 2004; Annesi, 2005).

#### 2.2.6 Nasa TLX

The Nasa TLX is an evaluative instrument of the workload referred to a specified task (Hart and Staveland, 1988). The questionnaire evaluates six items: mental demand, physical demand, temporal demand, effort, performance and frustration. Every item is described in an informative schedule given to the participant during the compilation of the questionnaire as a reminder. In the first part of the questionnaire, the six items are paired in all possible combinations (e.g., frustration vs. effort, temporal demand vs. performance). Participants must choose and circle the item which better describes the task (the MF or the NS) for each of the paired items proposed. The evaluator then records the recurrence of each item (weight). In the second part of the questionnaire, each item is proposed with a visual scale divided into 20 segments representing the workload, from “very low” to “very high.” For each of the six items, participants are requested to mark the position that better addresses the perceived specific load. The evaluator records the values given to each item (raw ratings). Subsequently, an adjusted rating (AR) is calculated for each item: AR = weight * raw rating. Finally, the overall workload is assessed using the formula: *Overall workload* = *(Σ AR)/15*.

#### 2.2.7 Cubo Fitness Test

The Cubo Fitness Test (CFT) is an evaluation instrument of motor efficiency (Invernizzi et al., 2021, 2022) composed of 5 submaximal tests performed on multiple functions cube-shaped modular tool: the Ruffier’s test (RUT), the 30s push-up test (PUT), the 30s sit-up test (SUT), the shoulder mobility test (SMT) and the chair sit and reach test (SRT). RUT measures cardiorespiratory fitness, the PUT and the SUT muscular fitness, while the SMT and the SRT flexibility. All the tests are executed at moderate perceived intensity and evaluated with perception scales such as the adapted Borg’s CR-10 scale (Rikli and Jones, 2013) or the Stretching Intensity Scale (Freitas et al., 2015) specifically developed for the stretching exercises. The Borg’s CR-10 scale has been used to evaluate RUT, PUT and SUT while SMT and SRT (which assess the flexibility fitness) has been evaluated with the Stretching Intensity Scale. At the end of the test battery, a final index of motor efficiency (IME) is calculated. The entire protocol of the CFT has been widely described in previous publications (Invernizzi et al., 2021, 2022).

### 2.3 Data analysis

The Shapiro–Wilk test was performed with skewness and Kusrtosis analysis to assess the normal distribution of the data. The ANOVA 2×2 analysis (time × group) was performed to evaluate the difference between pre- and post- the two experimental stimuli (MF and NS) in the total score and the TMD of the ITAMS questionnaire. The effect sizes for the ANOVA 2 × 2 were calculated as partial eta squared (ɳ^2^p), using the small = 0.02, medium = 0.13 and large = 0.26 interpretation for effect size. The T-test for dependent means was performed to investigate the differences in CFT performance after the two stimuli (MF and NS) while, to analyze the Nasa TLX data, was used the Wilcoxon test due to a violation of the normal distribution of data. In all tests, the significance level was set at α = 0.05. The effect size for the *T*-test was assessed using Cohen’s *d*. The interpretation of the effect size was performed using the following cut-off: 0.2 = “small” effect size, 0.5 = “medium” effect size and 0.8 = “large” effect size.

### 2.4 Results of study 1

**Figure 1:**
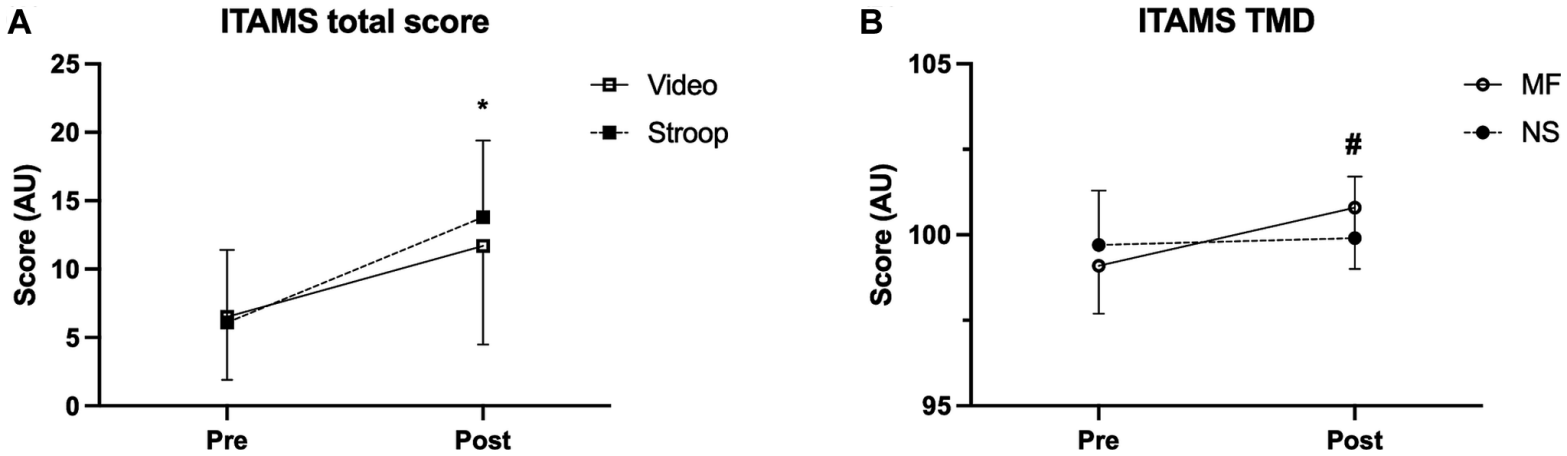
ITAMS questionnaire outcomes: interactions of the ITAMS total scores (panel **A**) and ITAMS Total mood disturbance scores (panel **B**). Data are expressed as arbitrary unit (au). NS = neutral stimulus, MF = mental fatigue stimulus. * = time effect (*p* < 0.05). # = significant interaction (*p* < 0.05).

The IPAQ confirmed that students recruited were sedentary, with a weekly amount of 532.6 ± 58.4 METs. The results of ITAMS’ total score and TMD at pre-stimulus did not show any significant difference between conditions (ITAMS total score: *p* = 0.476, Confidence interval: lower = −0.632, upper = 1.30; TMD: *p* = 0.077, Confidence Interval: lower = −1.30, upper = 0.074). The analysis of the total score of the ITAMS questionnaire (Figure 1A) evidence a tendency to significance in the interaction (*F* = 4.384, *p* = 0.052, ɳ^2^p = 0.205) and a significant time effect (*F* = 45.291, *p* < 0.0001, ɳ^2^p = 0.727) while no group effect was highlighted (*F* = 3.728, *p* = 0.070, ɳ^2^p = 0.180).

Concerning TMD (Figure 1B), the MF resulted in more impactful for participants’ moods as evidenced by the significant interaction (*F* = 7.960, *p* = 0.012, ɳ^2^p = 0.319) and a significant time effect (*F* = 7.020, *p* = 0.017, ɳ^2^p = 0.292).

Considering the Nasa TLX results (Table 2), differences between MF and NS were found in “Temporal Demand” (*p* = 0.036; *d* = −0.77 moderate) and in “Frustration” (*p* = 0.001; *d* = 0.41 small). No differences between MF and NS were detected in CFT outcomes (Table 3).

**Table 2:** Results of Nasa TLX questionnaire.

Items	MF	NS	*p*-value	Cohen’s d
Mental demand (au)	59.6 ± 23.7	51.3 ± 24.4	0.157	−0.35
Physical demand (au)	2.5 ± 7.4	3.6 ± 6.9	0.601	0.15
Temporal demand (au)	32.1 ± 22.9^*^	15.9 ± 18.8	0.036	−0.77
Performance (au)	21.2 ± 18.1	15.1 ± 19.2	0.181	−0.33
Effort (au)	46.2 ± 21.2	38.1 ± 22.6	0.158	−0.37
Frustration (au)	30.5 ± 28.8^*^	42.1 ± 28.4	0.001	0.41
Weighted sum (au)	12.8 ± 1.2	11.3 ± 2.0	0.349	−0.91

Results are expressed as mean ± standard deviation.

* Difference between mental fatigue (MF) and neutral (NS) stimuli, *p* < 0.05.

**Table 3:** Cubo Fitness Test (CFT) performance after Mental fatigue and Neutral stimuli.

CFT tests	MF	NS	*p*-value	Cohen’s d
Ruffier’s test (au)	24.2 ± 7.0	25.3 ± 7.8	0.276	0.15
30s Push-up Test (au)	8.7 ± 4.5	8.1 ± 4.2	0.311	−0.14
30s Seated sit-up test (au)	11.5 ± 4.7	12.0 ± 4.4	0.376	0.11
Shoulder mobility (cm)	49.0 ± 7.2	48.8 ± 8.2	0.692	−0.13
Chair sit and reach (cm)	0.1 ± 10.3	−0.2 ± 9.8	0.691	−0.03
Index of motor efficiency (au)	51.7 ± 13.5	52.0 ± 13.5	0.789	0.02

Au = arbitrary unit. The confidence intervals (95%) are referred to the difference between Mental fatigue (MF) and Neutral (NS) stimuli.

### 2.5 Summary of study 1

Individual CFT performance do not differ after receiving mental fatiguing or neutral stimuli before testing. We can assume that CFT does not present internal responsiveness to mental fatigue.

## 3 Study 2

Study 2 aims to examine at which intensity of perceived exertion (*weak*, *moderate*, *strong*, or *absolute maximum*) the CFT presents the higher relative reliability for external (performance) and internal (perceived exertion) loads. CFT was performed twice at different intensities of perceived exertion. At the end of the performance, the CFT’s workload characteristics were evaluated through the Nasa TLX questionnaire surveyed after testing.

### 3.1 Methods of study 2

Twenty-four (weight: 69.0 ± 8.6 Kg; height: 172.7 ± 10.0 cm; BMI: 23.1 ± 2.1 kg/m2) sedentary university students of 20.7 ± 1.7 years old were recruited for the second study (16 males and eight females). None of the participants partook in study 1. Inclusion criteria were belonging to sedentary university students who spent at least 7 h/day studying or attending university lessons. Students practicing regular sports or physical activity during free time were excluded. The main analysis performed in study 2 is based on the Intraclass correlation coefficient (ICC; Liaw et al., 2008). Hence, the sample size and the expected number of repeated measures can be considered reliable with an ICC minimum value of 0.6 (Bujang and Baharum, 2017). As for study 1, study 2 was conducted in accordance with the declaration of Helsinki and was approved by the ethics committee of the University of Milan (14 September 2020, number 84/20).

### 3.2 Procedure

Participants were recruited after administering the IPAQ questionnaire. The CFT was executed in different modalities, which were specifically differentiated by requiring participants to perform at intensities corresponding to the *weak*, *moderate*, *strong*, or *absolute maximum* verbal anchors of Borg’s CR-10 scale of perceived exertion. Consequently, the CFT protocol was modified to fit with the different modalities proposed, as follows:

#### 3.2.1 Cardiorespiratory fitness (CRF)

The cardiorespiratory fitness test is an adaptation of the Ruffier test. Participants were required to sit and stand from the sitting part of the cube with a self-paced rhythm reaching the required perceived exertion (*weak*, *moderate*, *strong*, or *absolute maximum* on Borg’s CR-10 scale) in 45 s. Every 10 s the evaluator gives feedback about the elapsed time to permit the participant to adapt the rhythm to better address the effort.

#### 3.2.2 Muscular fitness

For each of the two tests (PUT = 30s push-up and SUT = 30s seated sit-up), participants had 30 s to reach the required perceived exertion (*weak*, *moderate*, *strong*, or *absolute maximum* on Borg’s CR-10 scale), choosing from the three possible difficulty levels as described in Study 1.

#### 3.2.3 Flexibility fitness

Participants were asked to stretch for each test (SMT = shoulder mobility and SRT = chair sit and reach), performing an effort comparable to the required perceived exertion (*weak*, *moderate*, *strong*, or *absolute maximum* on Borg’s CR-10 scale). For study 2, to determine the stretching intensity, the Borg’s CR-10 scale was used instead of the SIS scale. This choice was made in order to equalize the intensity of flexibility tests to the cardiorespiratory and muscular fitness tests (weak, moderate, strong, and absolute maximum; Laur et al., 2003; López-de-Celis et al., 2022).

Before the beginning of the experiment, participants accomplished 3 days of familiarization with the perception scales and CFT. Successively, the participants performed the CFT twice (test and retest) at each intensity (*weak*, *moderate*, *strong*, or *absolute maximum*; Liaw et al., 2008). The necessary eight testing days to complete all measurements were distanced 1 week, one from another. The test calendar (comprising the order of the intensities proposed on the different days) was randomized using the site www.randomization.com to avoid unwanted side effects caused by the sequence of the perceived exertions. During each test day, before the CFT, participants were requested to express the value which better addressed their psychophysical recovery with the TQR scale (values up to 13 were considered adequate to let the participants begin the test session) and then to perform the CFT test at the programmed modality. To better understand the characteristics of the proposed workload, at the end of each test session, participants estimated their tasks with the Nasa TLX.

### 3.3 Data analysis

The test–retest reliability of the CFT proposed at different perceived exertion was investigated using the Intraclass correlation coefficient (ICC; Liaw et al., 2008). The ICC was assessed for the test performance (CFT performance values) and the perceived exertion values provided by participants (RPE perception values). Given the sample size of Study 2 and the number of measurements performed, the ICC values higher than 0.6 with a value of p lower than 0.05 were considered reliable (Bujang and Baharum, 2017). Moreover, the One-Way analysis of variance (ANOVA) was performed to verify the differences in workload (Nasa TLX), intensities perceived and CFT results at different modalities, using the mean obtained with the values of test and retest measures. The post-hoc test was performed with the Bonferroni correction. An α = 0.05 was considered for this analysis.

### 3.4 Results of study 2

The IPAQ confirmed that students recruited were sedentary, with a weekly amount of 628.4 ± 58.4 METs.

The CFT performances were found reliable in all the modalities proposed except for the *weak* modality in CRF and in the Index of Motor Efficiency (IME), and for the *absolute maximum* modality in CRF (Table 4). The values of RPE perceptions given by participants were found to be reliable in all CFT tests proposed at *moderate* and *strong* modalities, while the *weak* and *absolute maximum* modalities did not meet the reliability for any of the CFT tests. All RPE ICC values are reported in Table 4.

**Table 4:** Intraclass Correlation Coefficient (ICC) of the CFT (performance) and RPE (perception) results at different intensities.

	Modalities	CRF	PUT	SUT	SMT	SRT	IME
CFT (performance results)	Weak	0.014	0.876^*^	0.808^*^	0.960^*^	0.909^*^	0.147
	Moderate	0.805^*^	0.944^*^	0.823^*^	0.943^*^	0.838^*^	0.655^*^
	Strong	0.810^*^	0.944^*^	0.852^*^	0.984^*^	0.939^*^	0.518^*^
	Absolute maximum	0.413	0.955^*^	0.755^*^	0.985^*^	0.919^*^	0.843^*^
RPE (perception results)	Weak	−0.603	−0.852	−0.793	0.093	−0.28	−0.433
	Moderate	0.642^*^	0.737^*^	0.740^*^	0.690^*^	0.723^*^	0.831^*^
	Strong	0.810^*^	0.765^*^	0.758^*^	0.788^*^	0.846^*^	0.822^*^
	Absolute maximum	−0.459	−0.105	0.405	−0.018	−0.111	0.423

Data refers to ICC r values. CFT = Cubo Fitness Test, RPE = Rate of perceived exertion by CR-10 Borg’s scale. CRF = cardiorespiratory fitness, PUT = 30s push up test, SUT = 30s seated sit up test, SMT = shoulder mobility test, SRT = seated sit and reach test, IME = index of motor efficiency.

* *p* < 0.05.

Test–retest detailed results of CFT performances and of RPE perceptions are provided in Supplementary Table 1.

The one-way ANOVA analysis showed differences in the CFT tests’ performances for most of the modalities proposed, while no differences were detected in *strong* and *absolute maximum* modalities in the CRF, SUT, SMT, SRT and IME tests (RUT: *p* = 1.000; SUT: *p* = 0.051; SMT: *p* = 1.000; SRT: *p* = 1.000). Moreover, a similarity between the results of *weak* and *moderate* modalities was found in RUT (*p* = 1.000), SMT (*p* = 0.119) and SRT (*p* = 0.080). A similarity between the results of *moderate* and *strong* modalities was found only for SMT (*p* = 0.253) and SRT (*p* = 0.725.) The RPE perception values differed for each modality considered (*p* < 0.05). The mean Test results and mean perception values of each modality are reported in Supplementary Table 2.

**Figure 2:**
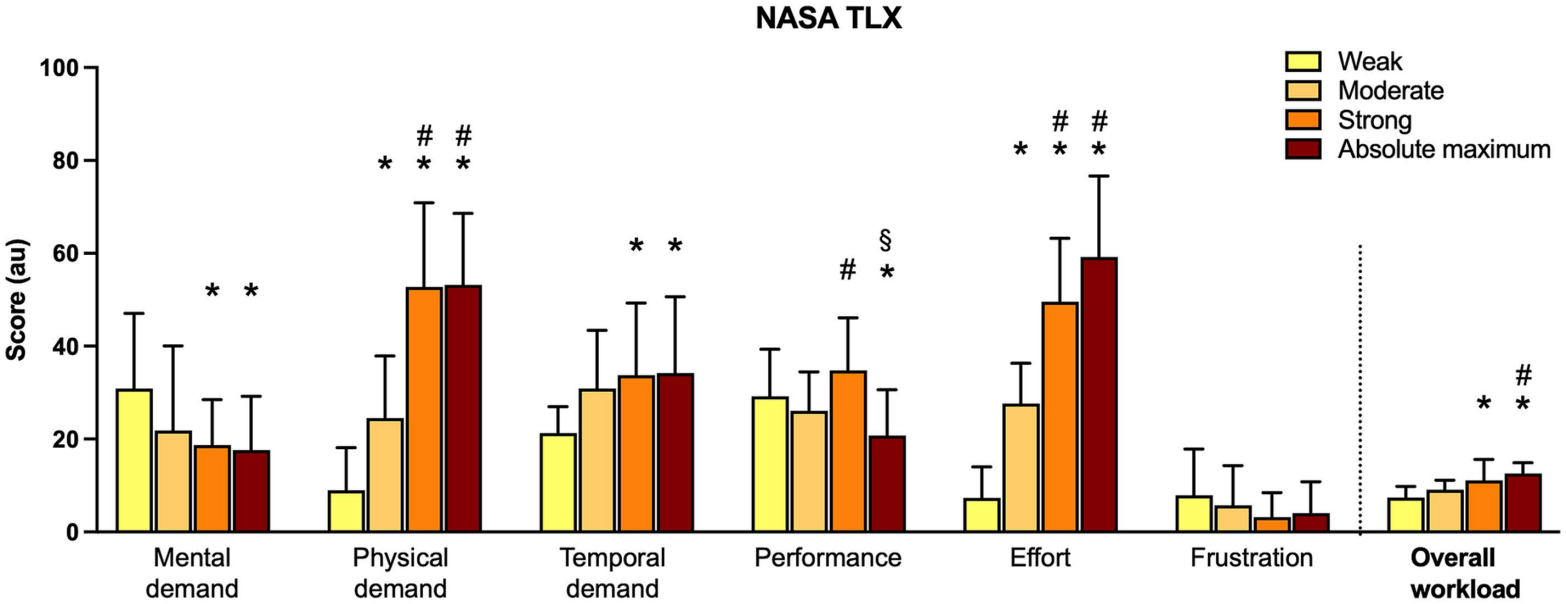
NASA TLX outcomes of domains (Mental demand, Physical demand, Temporal demand, Performance, Effort, and Frustration) and Overall Workload. Significant differences: * = against *weak* (*p* < 0.05), # = against *moderate* (*p* < 0.05), § = against *strong* (*p* < 0.05).

The one-way ANOVA analysis performed on the overall workload of the Nasa TLX outlined significant differences. The *post hoc* test outlined that the *weak* modality was different from the *strong* and *absolute maximum* modalities, while the *absolute maximum* modality differed from *moderate* (Figure 2). The complete results of the Nasa TLX are reported in Figure 2.

### 3.5 Summary of study 2

CFT performance values present relative reliability at different intensities except in CRF and IME domains when performed at *weak* intensity and in the CRF domain at *absolute maximum* intensity. In this analysis, moderate and strong modalities present reliability in all tests performed. The RPE perception values are reliable in all CFT domains proposed at *moderate* and *strong* modalities. The overall workload from Nasa-TLX showed significant differences. The *weak* modality differs from *strong* and *absolute maximum* modalities and *absolute maximum* from *moderate*.

## 4 Discussion

This study investigated the internal responsiveness stating that the CFT must not be influenced by acute mental fatigue to be a valid instrument for assessing motor efficiency (study 1). Moreover, this study explored which intensity of perceived exertion among *weak*, *moderate*, *strong*, and *absolute maximum* could be more appropriate to submit the CFT to a population of sedentary university students (study 2).

### 4.1 Study 1

Results evidenced that the two stimuli proposed had a different impact on participants. Concerning the ITAMS (mood evaluation) total scores, both MF and NS reported a worsening in the mood state. Nevertheless, considering the total mood disturbance results, MF reported a significant variation differently than NS, which reported a significantly lower alteration (close to 0). Noticeably, Nasa TLX (task’s workload) suggested that a temporal demanding load characterizes MF, while NS is a more frustrating task for the participants.

Understanding whether mental fatigue is correctly induced represents a significant challenge. In the study of Pageaux et al. (2014), no mood alteration caused by the intervention was found, while with Nasa TLX, a significant increase in mental demand and effort was observed. In another similar study by Staiano et al. (2019), the BRUMS (mood evaluation) and Nasa TLX questionnaires were employed to evaluate the effect of mental fatigue on time trial kayak training. Like our protocol, they proposed two stimuli (mental fatigue stimulus with the Stroop colour word task and neutral stimulus with a documentary), showing mood alteration and a higher workload after the mental fatigue stimulus compared with the neutral. In both studies (Pageaux et al., 2014; Staiano et al., 2019), the results were considered adequate to define participants as mentally fatigued. Indeed, the increasing total mood disturbance and perceived workload seem related to mental fatigue, which is defined as a “psychobiological state caused by prolonged exposure to a challenging cognitive activity, where the subject experience feeling of tiredness and lack of energy” (Marcora et al., 2009). Given that, we point out that the participants of the study 1 experienced a worsening of their mood along with an increased perceived workload after MF, probably determining a mental fatigue status (Marcora et al., 2009; Pageaux et al., 2014; Pageaux and Lepers, 2016; Staiano et al., 2019).

However, despite the mental fatigue, CFT did not report a significant difference from the neutral condition. It follows that the MF did not alter CFT. This seems different from what is highlighted by literature (Marcora et al., 2009; Pageaux et al., 2014; Smith et al., 2016; Van Cutsem et al., 2017a; Grgic et al., 2022), which stated that mental fatigue could have an impact on perceived effort and performance (Trecroci et al., 2020). A possible explanation could be found in the CFT characteristics. The physical tasks requested by the CFT have a short duration and a submaximal intensity (perceived as moderate). Factors such as duration and intensity of the physical task seem relevant when the influence of mental fatigue on performance is considered. The review by Van Cutsem et al. (2017b) reported that mental fatigue typically influences prolonged efforts while not affecting tasks of short duration, such as high-intensity exercises, power and anaerobic performances. Only a few studies reported an absence of mental fatigue influence on physical performance. Martin et al. (2016) assert that a higher inhibition capacity and resistance to mental fatigue induced by training, as in professional athletes, could reduce the influence of mental fatigue on performance. However, the participants of this study were not professional athletes. According to Van Cutsem et al. (2017a), high expertise and inefficient mental stimulus are possible causes for the lack of influence of mental fatigue on performance. As previously seen, we can exclude the inefficiency of the protocol in inducing mental fatigue. Hence, from the results reported by the literature, we could associate the lack of difference in CFT after a mental fatigue stimulus with the reduced time of effort (never more than 45 s), the low intensity of the proposed stimulus and the good familiarization of the participants with the proposed tests.

The obtained results are of particular interest for the CFT validation. Indeed, this test is strictly related to perceived exertion, and acute mental fatigue is demonstrated to influence effort perception, increasing it significantly (Marcora et al., 2009; Smith et al., 2016; Van Cutsem et al., 2017b). The ability of CFT to withstand mental fatigue can be a significant advantage for testing, mainly when implemented in university and desk-working environments. These environments often involve constant daily exposure to mentally draining stimuli (Mainsbridge et al., 2020). University students and desk workers perform in conditions of high temporal demand and, in some cases, can develop study and work-related frustration. All these conditions would not interfere with the CFT outcomes, giving an actual measure of the psychophysical status, even in those individuals already mentally exhausted (e.g., after or during intense studying and working days). The CFT aims to evaluate a wide range of sedentary populations also for being used even in universities or in working contexts as a fundamental element of the UP150 project (Invernizzi et al., 2021, 2022; Signorini et al., 2022). Having a submaximal test based on effort perception could represent an advantageous method to evaluate the motor efficiency of the students and workers regularly. The presence of an adequate evaluation in these populations could motivate improving psychophysical status with the help of structured intervention as for the UP150 project (Invernizzi et al., 2021, 2022; Signorini et al., 2022) and active pauses for desk workers and students, respectively (Keating et al., 2022).

### 4.2 Study 2

The aim of study 2 was to investigate which intensity modality to perform could present higher reliability (in CFT performance and RPE perception values) and be more adequate for the CFT protocol in a population of sedentary students.

#### 4.2.1 CFT performance

Of note, the index of motor efficiency was not reliable only at the *weak* modality of the CFT. This result is influenced by the values obtained in the CRF, which did not meet the reliability at the *weak* and *absolute maximum* modalities of the CFT. However, the other four sub-tests (SUP, SUT, SMT and SRT) were reliable.

From these results, we can assert that the most reliable results were *moderate* and *strong*, as also confirmed by previous studies (Invernizzi et al., 2021, 2022).

These results are influenced by the cardiorespiratory test proposed. Indeed, this is the only test of the CFT that was not found reliable in the *weak* and *absolute maximum* modalities. This could be due to the nature of the test, which is based on the measure of the Heart Rate (HR) and a self-pace execution differently from other submaximal cardiorespiratory measurements, which provide a guided execution based on rhythm, power or rates per minute in CFT (Wang et al., 2006). Both the CFT modalities considered (*weak* and *absolute maximum*) referred to widely known and embodied experiences (Borg, 1982; Bloomfield and Dale, 2015). Indeed, in common experience, people have more familiarities and clear-cut ideas of their maximal and minimum potentialities, while the shades are more poorly defined (Borg, 1990). A clearer idea of the target to reach (minimum or maximum effort) could influence the rhythmic execution of the test. Participants, indeed, are free to increase or decrease the intensity of the exercise to fit more precisely with the intensity requested (the evaluator gave feedback about the time every 10 s). The different intensity execution could have impacted HR, changing according to the exercise rhythm and affecting the final rate related to the intensity portrayed in the last seconds. Hence, the reliability of the *weak* and *absolute maximum* CFT modalities could have been impaired.

It is interesting to notice that the trend of reliability of results in cardiorespiratory fitness results is similar to what found in all perceived exertion (that will be better explained in the following paragraphs), highlighting a link between perceived exertion and HR as expressed in literature (Borg, 1982).

#### 4.2.2 CFT’s RPE perception

As evidenced by statistical analysis, participants reported significant differences between each modality of the CFT experienced considering perceived exertion.

Most of the given values coincided with the requested perception, demonstrating a good participants’ comprehension of the instructions the evaluator gave (except for *absolute maximum*, which resulted in lower than requested). Nevertheless, not all the intensities were found reliable. It could indicate that some intensities of effort could be more affordable to be perceived or reached than others. More specifically, the intensities *moderate* and *strong* seem easier to reach and perceive and are more reliable. It could have several explanations. Different authors stated that the *weak* perceived exertion, even if commonly experienced, could be more difficult to discriminate precisely and is more influenced by psychological factors than physiological ones due to fewer changes induced by physical exercise, which impede the reaching of an adequate stimulus perception threshold (Rejeski, 1981; O'Sullivan, 1984; Razon et al., 2012).

Moreover, *absolute maximum* perceived exertion could be harder to reach, possibly due to the reduced time of the tests, which could be inadequate to reach a maximal effort for the most trained participants (Swart et al., 2009). Indeed, as explained in the methodology section, most of the CFT protocol tests had a time of execution of 45 s or lower, which resulted inadequate for the most trained participant.

A different explanation arises from the flexibility fitness tests (SMT and SRT) of the CFT; indeed, these tests did not present a time of execution. The lack of reliability of *weak* and *absolute maximum* modalities could find an explanation for the daily variability of muscular tone which could depend on the activity made the day before the test and on the emotive status (Wahlström et al., 2003; Masi and Hannon, 2008). Considering the perceptive nature of the test, a variation of the basal muscular tone could influence the perception of what is “easy” and what is “difficult” to reach, varying the perceptive outcomes. Furthermore, in the *weak* modality, the participants were asked to perform a light effort that could result in too low to reach an adequate stimulation threshold of the proprioceptive receptors (like muscles spindles, the Golgi tendon organs or skin and connective tissue receptors; Halata, 1988) to precisely discriminate the physical perceptions (Refshauge et al., 1995). Consequently, this lack of precision could have influenced the obtained results. Concerning the *absolute maximum* CFT modality, the high effort that went into the stretch of the muscles highly involved the pain mechanism, probably activating the stretch reflex differently (Matre et al., 1998). The activation or non-activation of this typology of reflex could change the muscular tone, which makes it easier or more difficult to reach maximal stretching perception (Matthews, 1991) due to the augmented muscular tone. This phenomenon could consequently increase the effort to reach individual standards (Jones, 1995). Hence, the increased difficulty could result in a higher effort perceived, widening the variability between the two perception measures, possibly caused even by an altered mood state (Berger et al., 2016).

#### 4.2.3 NASA TLX outcomes

As previously highlighted, the Nasa TLX analysis seems to support the higher reliability of *moderate* and *strong* CFT modalities. Lower perceived intensity of CFT modalities determined higher mental demand and lower physical demand in Nasa TLX. This is not surprising, as lower exertion intensities have a lower impact on physical effort and consequently on physiological changes effort induced. Hence, due to this low perturbance of physiological status, this type of effort requires more cognitive activity to perceive the intensity (O'Sullivan, 1984). Furthermore, the Nasa TLX outcomes could explain even the poor reliability of the *absolute maximum* CFT modality. Analyzing the *absolute maximum* modality outcomes of the Nasa TLX, few differences with *strong* are reported, similar to CFT outcomes (see paragraph 4.2.1). Hence, from the participant’s point of view, the load characteristics of the two modalities were similar. The “physical demand” variable did not report significant differences, evidencing that the required physical effort to reach the target did not differ between the two modalities (they were both very high).

In the same way, the variable “effort” (which describes the difficulty of reaching the given task) evidences a similarity between them. At least, “temporal demand,” as in the previous variables, shows similar characteristics for *strong* and *absolute maximum* modalities. A similar “temporal demand” (which describe the grade of temporal pressure given by the task) could indicate an inadequacy of the test in raising the physical request from *strong* to *absolute maximum* intensity, as the participant were not able to detect a difference between the two stimuli proposed (Pöppel, 1997).

The variable “performance” (which describes the grade of satisfaction of the participants about how they performed) gives another interesting insight. Indeed, at the *absolute maximum* modality, participants reported a significative lower level of performance compared with the *strong* modality, indicating a sort of failure in reaching the given instructions (indeed, the perceived exertion reported at the *absolute maximum* modality was lower than 9 (au) as seen in Supplementary materials 1, 2; Lowndes et al., 2020).

Analyzing the overall workload measured with the Nasa TLX, it seems to increase gradually with the intensity proposed. *Moderate* and *strong* have similar characteristics, while *weak* and *absolute maximum* are opposite. Results from Figure 2 suggest three workload levels in performing CFT: a low level corresponding to *weak* and *moderate* modalities, an intermediate level corresponding to *moderate* and *strong* modalities, and a high load level corresponding to *strong* and the *absolute maximum* modalities.

In brief, the CFT performance were found reliable for each CFT test only at the *moderate* and *strong* modalities. Similarly, the most reliable RPE perception values were found at *moderate* and *strong* modalities. Finally, the CFT at different intensities could be characterized by three macro load levels identified by the Nasa TLX: the intermediate level is composed of *moderate* and *strong* CFT modalities. Considering the health purpose of the test, it could be asserted that the intensity which better addresses the CFT is the *moderate* version, as proposed in the original protocol. Even if the *strong* modality could constitute a valid alternative modality, the choice of a conservative modality (*moderate*) makes the test accessible to a broader range of populations, including young to older and sedentary to active people (Pescatello, 2014).

Of note, for the correct execution of the CFT, a familiarization period based on perceived exertion scales is needed (Razon et al., 2012). The present study indicates that the *moderate* and *strong* efforts are easier to be interpreted than *weak* and *absolute maximum*, facilitating the perceiving approach to those individuals not accustomed to perceived exertion scales.

The perceived exertion could represent a valid tool both for evaluation and education. Teaching how to perceive could educate people to manage their bodies and adequately use physical exercise for health. Furthermore, educating on good practices and the proper use of the body increase autonomy and the sense of competence, which could bring positive transfer to people’s life as a result of the increasing motivation, as evidenced in many motivational theories (Deci and Ryan, 2000; Teixeira et al., 2012).

The gender proportion could represent a limitation of the present study. Indeed, in both studies, the male/female ratio was unbalanced in favour of males. Moreover, the absence in the literature of effective methods that can determine with precision if and how mental fatigue affects participants could make it hard to understand the efficacy of the methodology in administering mental fatigue. Indeed, it cannot be excluded that an extremely cognitively demanding whole-day activity may be able to alter the results of CFT (due to a higher amount of mental fatigue).

## 5 Conclusion

Our study seems to evidence that CFT does not present an internal responsiveness to cognitive load, confirming that it could not be affected by acute mental fatigue. Furthermore, our study indicates that CFT presents relative reliability only for moderate and strong intensities. Anyway, an initial familiarization period on all level of intensities before testing is essential to favour a more adherent comprehension of the perceived exertion as suggested by literature.

## Data availability statement

The raw data supporting the conclusions of this article will be made available by the authors, without undue reservation.

## Ethics statement

The studies involving humans were approved by Comitato Etico Università degli Studi di Milano. The studies were conducted in accordance with the local legislation and institutional requirements. The participants provided their written informed consent to participate in this study.

## Author contributions

GS: Writing – original draft, Writing – review & editing, Conceptualization, Data curation, Formal analysis, Methodology. RS: Writing – original draft, Writing – review & editing, Supervision. AB: Formal analysis, Writing – review & editing. GM: Writing – original draft, Writing – review & editing. MR: Writing – original draft, Writing – review & editing. AT: Data curation, Formal analysis, Writing – review & editing. PI: Conceptualization, Methodology, Supervision, Writing – original draft, Writing – review & editing.
