# Supplementary material for: Effects of cognitive load and different exercise intensities on perceived effort in sedentary university students: a follow up of the Cubo Fitness Test validation
Source: Front Psychol. 2023 Dec 8;14:1254767. doi: 10.3389/fpsyg.2023.1254767 (PMC10742639; doi:10.3389/fpsyg.2023.1254767)
Supplement: Supplementary file 1 [file Table_1.docx]

Supplementary material

**Supplementary Table 1: main Outcomes of CFT results and Perceived exertions**

| **Measure** | **Intensity** | **Ruffier** | | **30s push-up** | | **30s seated sit-up** | | **Shoulder Mobility** | | **Chair sit & reach** | | **IME** | |
| --- | --- | --- | --- | --- | --- | --- | --- | --- | --- | --- | --- | --- | --- |
|  |  | ***Test*** | ***Retest*** | ***Test*** | ***Retest*** | ***Test*** | ***Retest*** | ***Test*** | ***Retest*** | ***Test*** | ***Retest*** | ***Test*** | ***Retest*** |
| *CFT*  *(Performance results)* | Weak | 26.4±8.1 | 26.3±7.3 | 5.0±2.3 | 5.4±2.4 | 6.4±2.2 | 6.9±3.1 | 55.4±4.6 | 55.8±4.6 | -3.0±5.4 | -3.3±5.9 | 42.2±8.7 | 43.4±9.0 |
|  | Moderate | 25.4±5.8 | 25.7±5.6 | 8.4±3.4 | 8.5±3.5 | 10.4±3.1 | 9.9±3.0 | 51.6±5.8 | 52.4±5.2 | 0.3±5.0 | 0.5±5.7 | 50.1±6.7 | 49.8±5.4 |
|  | Strong | 19.8±8.3 | 21.2±7.7 | 12.4±3.8 | 12.8±4.0 | 14.9±1.8 | 14.8±2.6 | 48.8±5.7 | 48.9±6.2 | 2.5±4.8 | 2.7±3.8 | 53.8±9.3 | 55.3±9.2 |
|  | Maximum | 19.0±8.1 | 18.7±7.0 | 15.5±4.3 | 15.5±4.0 | 17.0±1.6 | 16.4±3.0 | 46.8±5.4 | 47.1±5.7 | 4.1±4.8 | 4.7±4.9 | 58.6±10 | 57.8±10.2 |
| *RPE*  *(Perception results)* | Weak | 1.2±0.7 | 1.3±0.5 | 1.2±0.6 | 1.2±0.5 | 0.9±0.7 | 1.2±0.5 | 1.2±0.6 | 1.1±0.6 | 0.9±0.6 | 1.0±0.6 | 1.1±0.4 | 1.1±0.3 |
|  | Moderate | 3.3±0.4 | 3.2±0.4 | 3.0±0.5 | 3.1±0.4 | 2.6±0.4 | 2.7±0.4 | 2.8±0.6 | 2.8±0.7 | 2.7±0.6 | 2.6±0.6 | 2.9±0.3 | 2.9±0.3 |
|  | Strong | 6.0±1.1 | 6.0±1.1 | 6.9±1.1 | 6.9±0.9 | 5.2±1.0 | 5.2±1.0 | 6.9±1.2 | 6.8±0.9 | 6.6±1.4 | 6.7±1.2 | 6.3±0.9 | 6.4±0.8 |
|  | Maximum | 7.8±1.5 | 8.0±1.3 | 8.0±1.4 | 8.6±1.6 | 7.0±1.5 | 7.2±1.8 | 8.2±1.2 | 8.2±1.7 | 8.4±1.6 | 8.5±1.4 | 7.9±0.9 | 8.1±1.0 |

Data are expressed as mean ± standard deviation. All values are in arbitrary units (au) except for CFT's Shoulder mobility and chair sit & reach, which are in cm. IME= Index of motor efficiency
