# Supplementary material for: Effects of cognitive load and different exercise intensities on perceived effort in sedentary university students: a follow up of the Cubo Fitness Test validation
Source: Front Psychol. 2023 Dec 8;14:1254767. doi: 10.3389/fpsyg.2023.1254767 (PMC10742639; doi:10.3389/fpsyg.2023.1254767)
Supplement: Supplementary file 2 [file Table_2.docx]

Supplementary material

**Supplementary Table 2: Means of the test and retest results of the CFT (Performance) and RPE (Perception)**

| **Measure** | **Test** | **Weak** | **Moderate** | **Strong** | **Absolute Maximum** |
| --- | --- | --- | --- | --- | --- |
| CFT  *(Performance results)* | Ruffier (au) | 26.4 ± 5.5 | 25.6 ± 5.2 | 20.5 ± 6.1 *# | 18.9 ± 7.0 *# |
|  | 30s push-up (au | 5.2 ± 2.2 | 8.5 ± 3.3 * | 12.6 ± 3.8 *# | 15.5 ± 4.1 *#§ |
|  | 30s seated sit-up (au) | 6.6 ± 2.5 | 10.2 ± 2.9 * | 14.8 ± 2.1 *# | 16.7 ± 2.2 *# |
|  | Shoulder Mobility (cm) | 55.6 ± 4.5 | 52.0 ± 5.3 | 48.8 ± 5.9 * | 46.9 ± 5.5 *# |
|  | Chair sit & reach (cm) | -3.1 ± 5.4 | 0.4 ± 5.0 | 2.6 ± 4.2 * | 4.4 ± 4.7 *# |
|  | IME (au) | 42.8 ± 6.5 | 49.9 ± 5.2 * | 54.5 ± 7.6 * | 58.2 ± 9.4 *# |
|  |  |  |  |  |  |
| RPE  *(Perception results)* | Ruffier (au) | 1.2 ± 0.4 | 3.2 ± 0.4 * | 6.0 ± 1.0 *# | 7.9 ± 0.9 *#§ |
|  | 30s push-up (au) | 1.2 ± 0.3 | 3.0 ± 0.4 * | 6.9 ± 0.9 *# | 8.3 ± 1.0 *#§ |
|  | 30s seated sit-up (au) | 1.1 ± 0.4 | 2.7 ± 0.4 * | 5.2 ± 0.9 *# | 7.1 ± 1.3 *#§ |
|  | Shoulder Mobility (au) | 1.1 ± 0.4 | 2.8 ± 0.5 * | 6.8 ± 1.0 *# | 8.2 ± 1.0 *#§ |
|  | Chair sit & reach (au) | 0.9 ± 0.5 | 2.7 ± 0.5 * | 6.6 ± 1.2 *# | 8.4 ± 1.0 *#§ |
|  | IME (au) | 1.1 ± 0.2 | 2.9 ± 0.3 * | 6.3 ± 0.8 *# | 8.0 ± 0.7 *#§ |

Data are expressed as mean ± standard deviation. IMI= Index of motor efficiency. (au) = arbitrary unit. * = different than *weak* (p<0.05), # = different than *moderate* (p<0.05), § = different than *strong* (p<0.05.
